# Automatically Generated Datasets: Present and Potential Self-Cleaning Coating Materials

**DOI:** 10.1038/s41597-024-02983-0

**Published:** 2024-01-31

**Authors:** Shaozhou Wang, Yuwei Wan, Ning Song, Yixuan Liu, Tong Xie, Bram Hoex

**Affiliations:** 1 School of Photovoltaic and Renewable Energy Engineering, University of New South Wales, Kensington, NSW Australia; 2 GreenDynamics Pty. Ltd, Kensington, NSW Australia; 3 Department of Linguistics and Translation, City University of Hong Kong, Kowloon Tong, Hong Kong China

**Keywords:** Materials for devices, Materials for energy and catalysis, Materials for optics

## Abstract

The rise of urbanization coupled with pollution has highlighted the importance of outdoor self-cleaning coatings. These revolutionary coatings contribute to the longevity of various surfaces and reduce maintenance costs for a wide range of applications. Despite ongoing research to develop efficient and durable self-cleaning coatings, adopting systematic research methodologies could accelerate these advancements. In this work, we use Natural Language Processing (NLP) strategies to generate open- and traceable-sourced datasets about self-cleaning coating materials from 39,011 multi-disciplinary papers. The data are from function-based and property-based corpora for self-cleaning purposes. These datasets are presented in four different formats for diverse uses or combined uses: material frequency statistics, material dictionary, measurement value datasets for self-cleaning-related properties and optical properties, and sentiment statistics of material stability and durability. This provides a literature-based data resource for the development of self-cleaning coatings and also offers potential pathways for material discovery and prediction by machine learning.

## Background & Summary

Outdoor self-cleaning coatings are drawing attention in a world becoming more urbanized and plagued by pollution and dirt. These innovative coatings are engineered to maintain surface cleanliness without requiring intensive active cleaning, providing an effective solution for preserving the integrity of various surfaces in outdoor settings. Outdoor self-cleaning coatings are crucial due to key benefits, i.e., they prolong the lifespan of surfaces by reducing dirt accumulation and corrosion^1^, offer environmental advantages by lessening reliance on water-intensive and chemically-harmful traditional cleaning, cut maintenance costs^2^, and minimize the level of air pollution containing compounds detrimental to human health and the environment^3^. Studies on self-cleaning coatings indicate that the primary properties governing their functionality are either hydrophilicity or hydrophobicity, which facilitate the efficient removal of dirt via rainwater, and photocatalytic activity, which aids in the decomposition of pollutants into gaseous or small particulate forms^4^. With a unique ability to retain cleanliness, outdoor self-cleaning coatings hold immense potential for large-scale applications such as building and transportation^5–7^. Moreover, they are particularly pivotal in the field of solar-related renewable energy generation, where the cleanliness of the light-absorbing surfaces of solar panels^4,8^ and solar thermal collectors^9,10^ directly impacts efficiency. While research towards creating effective, durable, and cost-efficient self-cleaning coatings for various applications is advancing steadily, these efforts could be greatly expedited by employing more systematic research methodologies or multidisciplinary knowledge integration, compared to the conventional approach of iterative material-component substitution.

The data-driven materials discovery using machine learning, namely Materials Informatics, has gained increasing prominence recently^11–13^. This approach leverages existing computational and experimental data in the principles of findability, accessibility, interoperability, and reusability (FAIR)^14^ to ensure high quality and machine-actionability. Computational data, inherently structured, can be readily used for machine learning and can be obtained from open-source databases like the Materials Project^15^ and NOMAD^16^. On the other hand, although these databases offer some empirical data, most of such data is embedded within the scientific literature. The task of harvesting and processing this data for machine learning is challenging, as it involves the manual extraction of unstructured data, necessitating substantial human resources with specialized knowledge^17^. However, recent advancements in Natural Language Processing (NLP) methods, including tools such as ChemDataExtractor^18,19^ and language models^20–22^, have enabled efficient extraction and processing of data from the scientific literature^23,24^. While some review articles have attempted to manually gather data on outdoor self-cleaning coating materials^4,25^, their domain-specific nature leads to a lack of comprehensiveness. As such, employing advanced NLP strategies to address this deficiency constitutes a worthy endeavour in this field.

The datasets we present are both open- and traceable-sourced, comprising literature data from 39,011 multi-disciplinary papers. Beyond papers on self-cleaning coatings, materials investigated for different applications in a wide range of disciplines also show significant potential for outdoor self-cleaning applications. For example, the hydrophilic or hydrophobic traits of some marine antifouling coatings make them promising for self-cleaning applications, and their engineered stability and durability for harsh marine environments underscore their outdoor suitability^26^. Given this insight, we collected literature data in two ways: (1) function-based datasets focusing on self-cleaning, and (2) property-based datasets which include self-cleaning-related properties, i.e., hydrophilic, superhydrophilic, hydrophobic, superhydrophobic, oleophobic, superoleophobic, omniphobic, amphiphobic, and photocatalytic. We collect data on oleophobic and superoleophobic coatings as such coatings are also promising in the outdoor self-cleaning function facilitated by rainfall. This is due to the fact that most oils exhibit lower surface tension values compared to water, which implies that if a coating possesses oleophobic or superoleophobic attributes, it will, by extension, also have hydrophobic or superhydrophobic properties^27^. Similarly, surfaces with omniphobic and amphiphobic properties are extraordinarily repellent to all kinds of liquids. As such, these surfaces inherently demonstrate hydrophobic or superhydrophobic characteristics. The inclusion of literature data pertaining to these coatings will augment the repository of potential self-cleaning materials.

**Fig. 1:**
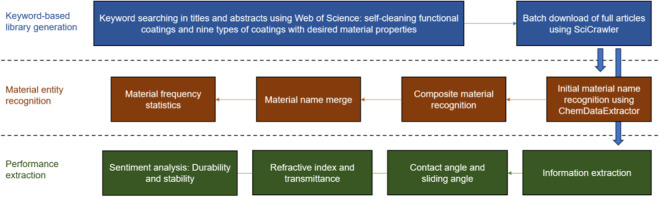
Extraction pipeline of datasets. The arrows indicate the workflow.

The datasets in this work offer refinement in literature data, enhancing training quality while minimizing the computational expenses associated with machine learning for self-cleaning material discovery. Concurrently, these datasets can function as a validation mechanism for such machine-learning outcomes. The datasets are presented in four formats for different potential uses: (1) frequency statistics datasets for the material name (43,571 materials), which can be used as a benchmark or an evaluation index in material discovery using unsupervised machine learning^28^; (2) material name dictionaries comprising material names, formulas, and corresponding acronyms, which is a pivotal resource for the standardization of material nomenclature in machine learning; (3) material measurement value datasets (16,420 data points) comprising water-related measurements (contact angle and sliding angle) and optical properties and performance (refractive index and transmittance), which can be employed in the manual discovery of self-cleaning or transparent self-cleaning materials, as well as in NLP tasks like material performance prediction after certain processing^21,22^; (4) statistics datasets for sentiment classification of material stability and durability (24,789 subjective sentences), which can be used in further opinion mining for material practicability prediction^29,30^. In this work, all data are fully traceable, with Digital Object Identifiers (DOIs) of the source publications provided for reference. Figure 1 illustrates the extraction pipeline of the datasets.

## Methods

This section provides the pipeline and methodologies implemented to produce the datasets, including the corpora creation, material name processing, dictionary formation, and data extraction.

### Corpus generation

A corpus comprising 39,011 articles was amassed from articles published by Elsevier within the date range of January 1, 2000 to December 31, 2022. The metadata of these articles were sourced from the Web of Science (WoS) using an advanced search feature. This search was executed utilizing specific keywords, as stipulated in Table 1, as indices for the subject matter within the article titles and abstracts. Under the publisher’s permission and in compliance with all relevant terms and conditions of the publisher, full texts of these articles were then retrieved via Elsevier’s Application Programming Interface (API) using SciCrawler^29^ in six dataset tags: SelfCleaning (consisting of 2,044 articles on self-cleaning coatings), Hydrophilic (with 8,921 articles on hydrophilic and superhydrophilic coatings), Hydrophobic (comprising 13,677 articles on hydrophobic and superhydrophobic coatings), Oleophobic (encompassing 581 articles on oleophobic and superoleophobic coatings), Omniphobic (involving 143 articles on omniphobic and amphiphobic coatings), and Photocatalytic (comprising 13,437 articles on photocatalytic coatings). It should be noted that there may be instances of duplicate counts within these six datasets due to the potential overlap of domains. The articles were then converted from the XML format to the text format. Subsequent preprocessing procedures entailed the removal of information not related to the desired datasets, including author names, affiliations, and references.

**Table 1:** Keywords used in Web of Science searching for each dataset.

Dataset tag	Function/Property	Descriptor
SelfCleaning	‘self-cleaning’	‘coating’ or ‘film’
Hydrophilic	‘hydrophilic’ or ‘superhydrophilic’	‘coating’ or ‘film’
Hydrophobic	‘hydrophobic’ or ‘superhydrophobic’	‘coating’ or ‘film’
Oleophobic	‘oleophobic’ or ‘superoleophobic’	‘coating’ or ‘film’
Omniphobic	‘omniphobic’ or ‘amphiphobic’	‘coating’ or ‘film’
Photocatalytic	‘photocatalytic’	‘coating’ or ‘film’

### Material name and dictionary processing

ChemDataExtractor^18,19^ was used to extract material names, including English terminology and corresponding abbreviations such as formulae and acronyms, for each designated category. Two dictionaries were subsequently created. The first, a sentence dictionary, was constructed to store the original sentence and any contained material names along with their respective starting and ending character indices. The second, a name-abbreviation pair dictionary, was formulated to store the pairs of material names and their abbreviations, and their associated paper DOIs. The example below shows the extraction of ‘TiO_2_’ and ‘Titanium dioxide’ pairs:

In cases where two extracted material names were linked via specific punctuations such as hyphens, colons, slashes, or dashes, these names were consolidated into a single entry as a composite material.

These six name-abbreviation pair dictionaries were then merged to create an expansive dictionary of abbreviations. A frequency threshold of greater than five was employed as a filter to preserve reliable abbreviations and their corresponding English names in the final pair dictionary. The sentence dictionary was then updated, replacing material names with their abbreviations. It should be highlighted that there may be unavoidable instances where some organic materials share the same abbreviations. Furthermore, it is important to note that not only target materials (i.e., functional materials) were recorded, but also recipe materials and potential by-products were documented within the process. The example below illustrates a final dictionary of ‘TEOS’ where the total material name frequencies in the literature are also shown:

### Material measurement value extraction and pairing

Sentences containing the target phrases, values, and material names were initially selected based on established criteria. Within these selected sentences, when a single material name and value are identified, they are directly paired. In instances where multiple material names are detected, a structured matching algorithm is employed. This process begins with the utilization of regular expressions to execute fuzzy matching based on predefined patterns, such as ‘[value] for [material]’ or ‘[material] (value)’. In the absence of discernible pairs, the sentence is segmented using punctuation marks and conjunctions, followed by an attempt to identify value-material pairs within these fragments. If pairs remain undetected, a dependency relationship is employed, wherein all-layer head words of each value and material name (encompassing both direct and indirect heads) are documented. If there’s an overlap in the head words for both the value and material name within certain layers, they are designated as a match.

The method for extracting contact and sliding angles involved filtering sentences that contained either or both of these phrases and the symbol “°”. Regular expressions, specifically the pattern *[0*–*9.]*+*[*±]*[0–9.]*°, were utilized to extract the corresponding angular values. This was conducted with the focus on retrieving absolute values only, thereby discarding any sentences that included comparisons such as ‘increase of’ or ‘lower’. If a plus-minus symbol (±) was present, only the value preceding this symbol was extracted. In some instances, both angles were mentioned within a single sentence (e.g., “The sliding angle and contact angle of TiO_2_ coating were 5° and 160° respectively.”). In such cases, a matching algorithm was devised that leveraged the positions of the keywords and dependency relationship within the sentence to associate each angle with its respective value. Contact angles of 50, 90, and 150 degrees were omitted due to their frequent use in defining hydrophilic and hydrophobic properties, thus not providing specific material characteristics. It should be noted that despite the role of rainfall in self-cleaning processes, we have refrained from extracting data for only ‘water contact angle’ and ‘water sliding angle’. This is due to the fact that certain studies, such as those involving oleophobic coatings, often employ organic liquids in their experiments instead of water.

For the refractive index, sentences containing the phrase ‘refractive index’ were filtered. Only those sentences including ‘at … nm’ or ‘maximum’ were retained. The regular expression pattern *[0*–*9]{1}[.]{1}[0*–*9]+* was applied to extract the refractive index value, focusing on absolute values and omitting sentences with comparative phrases. In the presence of a plus-minus symbol (±), only the first value preceding it was considered. The range of acceptable values was limited between 1 and 10.

For transmittance, sentences were filtered based on the presence of both ‘%’ and ‘transmittance’. As with the refractive index, only sentences including ‘at … nm’ or ‘maximum’ were retained. The regular expression pattern *[0*–*9.]*+*[*±|–]*[0–9.]*% was employed to extract the transmittance value, again focusing solely on absolute values and excluding sentences with comparative phrases. If a plus-minus symbol (±) or a dash (–) was present, only the initial value before the symbol was extracted. The permissible range of transmittance values was restricted between 90 and 100. Although the extinction coefficient, a normalized property, is a more scientific term, it is relatively rarely reported compared to the thickness-dependent transmittance. However, when the thickness is known, transmittance serves as a practical measure to facilitate comparative analyses of materials.

### Durability and stability

The method for evaluating durability and stability entailed a multi-step process. For each category, sentences were filtered based on the presence of either ‘durability’ or ‘stability’ and a length of fewer than 500 characters. Following this, a convolutional neural network (CNN) sentiment analysis model^29,30^ pre-trained on energy-related text was utilized to extract sentences expressing an opinion, dividing these sentences into positive and negative categories based on the sentiment conveyed. Each material was then associated with its respective frequency of positive and negative sentiments. The positive rate was computed as the ratio of the frequency of positive sentiment to the total frequency (positive + negative), and this data was stored in a dictionary, enabling the ranking of materials based on their positive rate. For each appearance of a material name, classification result, and its DOI were recorded. The positive and negative sentiments were denoted as ‘driver’ and ‘barrier’ respectively.

## Data Records

The datasets presented in this work are available online in JSON format on Figshare^31^. In accordance with the publisher’s relevant terms and conditions, the datasets contain only data and do not include any excerpts from the original text. A detailed description of data records is provided in Table 2. These different formatting styles provide various perspectives on the data, enabling users to utilize the datasets effectively based on their specific research requirements.

**Table 2:** Description of file label and data key label in data records.

File Name or Feature Name	Description	Data Type
material_statistics	Material name statistics by frequency.	List
material_dictionary	Material name dictionary for English terminology, formulae and acronyms.	Dict
sentiment_statistics	Result of sentiment analysis for durability and stability, classified by ‘driver’ and ‘barrier’.	Dict
contact_angle	Numerical values of contact angle.	Float
sliding_angle	Numerical values of sliding angle.	Float
refractive_index	Numerical values of refractive index.	Float
transmittance	Numerical values of transmittance.	Float
doi	Digital object identifier of the paper.	String
freq	Frequency of the material name or the number of sentiments.	Int
driver	Positive sentiment with references.	String
barrier	Negative sentiment with references.	String

## Technical Validation

In this technical validation section, we will evaluate the chosen five coating properties for self-cleaning function, and also evaluate the data reliability of presented datasets manually and statistically.

### Property validation for self-cleaning

The validity of the properties under investigation, namely Hydrophilic, Hydrophobic, Oleophobic, Omniphobic, and Photocatalytic, as the key attributes for self-cleaning was ascertained by assessing their coverage over the SelfCleaning dataset. It was determined that the coverage of the full set of five property subsets was 58.1% and that of the noise-removed set (from which materials with a frequency of less than 10 were excluded) was 77.3% as shown in Table 3. Moreover, we also found that the inclusion of ‘Antisoiling’ as a self-cleaning property significantly improved the coverage of the full set to 94.7% and the noise-removed set to 97.8%. This suggests that the properties of Hydrophilic, Hydrophobic, Oleophobic, Omniphobic, Photocatalytic, and Antisoiling collectively constitute the comprehensive mechanisms for self-cleaning coatings. Nonetheless, it should be noted that antisoiling represents an independent and substantial area that is beyond the scope of this work.

**Table 3:** Material numbers and coverages of Hydrophilic, Hydrophobic, Oleophobic, Omniphobic, and Photocatalytic datasets on SelfCleaning dataset.

Dataset	Collected in property datasets	Total in SelfCleaning dataset	Coverage
Full dataset	2341	4026	58.1%
Noise-removed dataset	1553	2009	77.3%

### Manual validation

A manual evaluation was conducted to determine the dataset reliability by precision and recall metrics:

1 $$precision=\frac{TP}{TP+FP}$$

2 $$recall=\frac{TP}{TP+FN}$$

3 $${F}_{score}=\frac{precision\cdot recall}{precision+recall}\times 2$$

where true positive, TP, is the number of extracted correct data, false positive, FP, is the number of extracted incorrect data, and false negative, FN, is the number of omitted correct data.

To evaluate the material name extraction, 100 sentences were randomly selected from the corpora of each dataset for analysis. The correctness of extraction, signified by TP, FP and FN, was determined through the comparison of automatic extraction results and the expert’s extraction results. Table 4 provides a summary of the evaluation outcomes. The precision of extraction is generally observed to be high across the six datasets, however, recall rates appear to be comparatively low. The lower recall can be attributed to infrequently used material names and abbreviations in coatings, particularly for organic or composite materials, which may not be incorporated in ChemDataExtractor (a tool primarily geared towards materials encountered in natural science). Particularly, the Hydrophilic, Hydrophobic, Oleophobic, and Omniphobic datasets exhibit low recall, this can be attributed to the multidisciplinary nature of these datasets spanning fields such as biology and medicine. For example, hydrophilic, hydrophobic, oleophobic, and omniphobic properties significantly impact the interaction between drugs, cells, and biological membranes, and thus these properties are widely discussed in medicine domains.

**Table 4:** Precision, recall and F_score_ of material name datasets.

Dataset	TP	FP	FN	Precision	Recall	F_score_
SelfCleaning	33	4	6	89.2%	84.6%	86.8%
Hydrophilic	48	3	13	94.1%	78.7%	85.7%
Hydrophobic	35	3	9	92.1%	79.6%	85.4%
Oleophobic	33	9	11	78.6%	75.0%	76.7%
Omniphobic	31	6	10	83.8%	75.6%	79.5%
Photocatalytic	32	7	7	82.1%	82.1%	82.1%
Total	212	32	56	86.9%	79.1%	82.8%

Precision assessments for the contact angle, sliding angle, refractive index, and transmittance data are delineated in Tables 5–8. The extraction of these characterization datasets was evaluated by manually reviewing 100 randomly selected sentences containing the target characterization (100 sentences for each characterization) from SelfCleaning dataset and each applicable property dataset. Several datasets either lacked specific measurement results, were not applicable for certain measurements, or contained insufficient sentences with target results. Contact and sliding angle measurements are not relevant for the Photocatalytic dataset, and sliding angle measurements do not apply to the Hydrophilic dataset. Furthermore, as we applied relatively strict data extraction criteria, the Omniphobic dataset only includes 9 sliding angle data points, and the Oleophobic dataset comprises merely 4 refractive index data points, while the Omniphobic dataset does not contain any refractive index results. Additionally, the datasets for four properties have limited transmittance results, with 46 data points in Hydrophilic, 4 in Oleophobic, 3 in Omniphobic, and 48 in Photocatalytic datasets. The evaluation of recall was not performed for these characterization extractions, given their considerably lower frequency in the literature relative to material names. Despite the undetermined recall rate for data extraction, the quality of the data can be assured owing to the high precision of these four characterization extractions. The overall precision for each characterization is computed by summing the total TP and total FP.

**Table 5:** Precision of contact angle extraction.

Dataset	TP	FP	Precision
SelfCleaning	87	13	87.0%
Hydrophilic	72	28	72.0%
Hydrophobic	74	26	74.0%
Oleophobic	70	30	70.0%
Omniphobic	76	12	86.0%
Total	379	109	77.7%

**Table 6:** Precision of sliding angle extraction.

Dataset	TP	FP	Precision
SelfCleaning	85	15	85.0%
Hydrophobic	77	23	77.0%
Oleophobic	26	6	81.3%
Omniphobic	6	3	67.0%
Total	194	47	80.5%

**Table 7:** Precision of refractive index extraction.

Dataset	TP	FP	Precision
SelfCleaning	91	9	94.0%
Hydrophilic	84	16	84.0%
Hydrophobic	79	21	79.0%
Oleophobic	3	1	75.0%
Photocatalytic	89	11	89.0%
Total	346	58	85.6%

**Table 8:** Precision of transmittance extraction.

Dataset	TP	FP	Precision
SelfCleaning	80	11	87.9%
Hydrophilic	40	6	87.0%
Hydrophobic	91	9	91.0%
Oleophobic	3	1	75.0%
Omniphobic	1	2	33.3%
Photocatalytic	44	4	91.7%
Total	259	33	88.7%

Even though durability and stability are critical attributes for outdoor coatings, the absence of standard measurements to appraise these properties necessitates a different approach. Consequently, we have extracted authors’ opinions from the literature to synthesize an overarching perspective on the materials in durability and stability. For the sentiment analysis evaluation, 100 sentences conveying subjective sentiments about material durability and stability from each dataset, along with their automated classification results, were randomly selected and manually assessed for accuracy. It should be noted that only 94 subjective sentences about material durability and stability were identified in the Omniphobic dataset due to the relatively smaller size of its corpus. The precision achieved in the sentiment analysis is notably high as shown in Table 9, highlighting the reliability of these data in durability and stability assessments.

**Table 9:** Precision of sentiment sentence classification for durability and stability.

Dataset	TP	FP	Precision
SelfCleaning	92	8	92.0%
Hydrophilic	88	12	88.0%
Hydrophobic	92	8	92.0%
Oleophobic	94	6	94.0%
Omniphobic	89	5	94.7%
Photocatalytic	92	8	92.0%
Total	547	47	92.1%

### Statistical validation

**Fig. 2:**
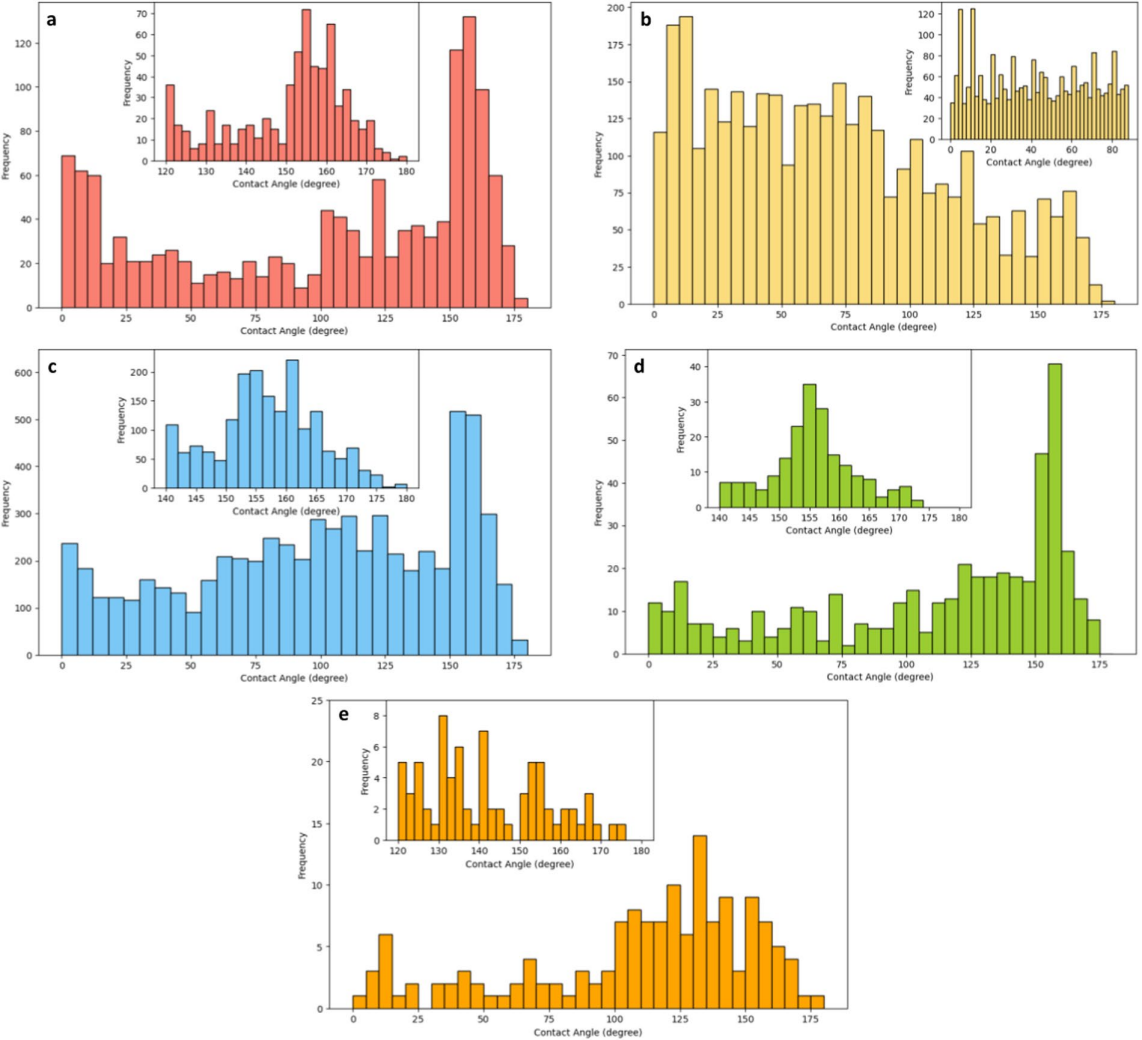
Contact angle distributions of (**a**) SelfCleaning, (**b**) Hydrophilic, (**c**) Hydrophobic, (**d**) Oleophobic and (**e**) Omniphobic datasets. The insets show the distributions in the high-frequency ranges.

Statistical assessments were executed to evaluate the reliability of the dataset. The distribution of contact angles generally aligns with the anticipated trends. Figure 2a illustrates the distribution of contact angles in the SelfCleaning dataset, predominantly occurring at lower values (<10 degrees) and higher values (>150 degrees). This distribution can be attributed to the fact that hydrophilicity and hydrophobicity are integral properties for self-cleaning. As a result, most research on self-cleaning coatings is dedicated to engineering surfaces with these extreme characteristics.

Figure 2b presents the distribution of contact angles in the Hydrophilic dataset, with the majority of values falling in the lower range and decreasing frequency as the contact angle increases. This deviates slightly from the anticipated distribution, where a significant concentration of values at the lower end and only a minimal scattering in the higher range were expected. This discrepancy can be accounted for the corpus design. For example, not only do papers studying hydrophilic coatings contain the keyword ‘hydrophilic’, but papers covering a range of measurements from small to large contact angles also employ ‘hydrophilic’ in the titles or the abstracts. Nevertheless, users have the discretion to exclusively select data from the lower range for applications.

Figure 2c,d depict the distribution of contact angles in the Hydrophobic, Oleophobic, and Omniphobic datasets, respectively. Predominantly, the angles fall within the higher value range, but a minor peak at the lower range is also visible. The explanation for this unexpected distribution pattern could be the same as for the Hydrophilic dataset, i.e., the keyword-based selection of papers might include studies that cover a wider range of contact angle measurements.

**Fig. 3:**
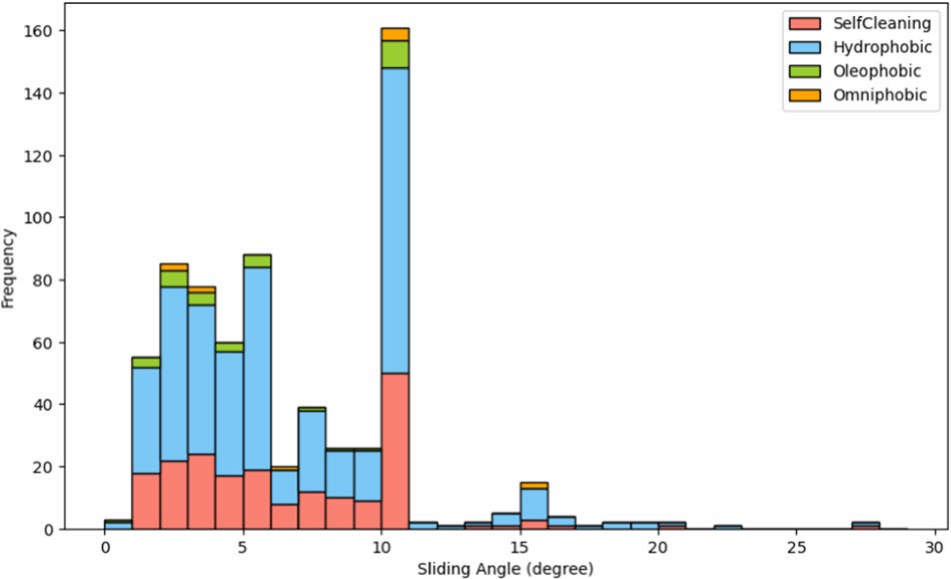
Total sliding angle distribution of SelfCleaning, Hydrophobic, Oleophobic and Omniphobic datasets. The portion of each dataset is shown.

Figure 3 exhibits the distribution of sliding angles for four pertinent datasets. The distribution follows the expected trend, with the majority of the frequency predominantly localized in the lower angle range (<10 degrees). Among the datasets, the Hydrophobic dataset accounts for the largest proportion of frequency, which is attributable to its largest corpora. A noticeably high frequency is observed at the 10-degree mark. This anomaly arises from the common practice of using 10 degrees as a generalized range for summarizing sliding angles, e.g., a statement ‘A sliding angle of less than 10° was measured for the … samples characterizing them as superhydrophobic surfaces.’ Nevertheless, such data noise can be easily rectified using algorithms prior to further data processing.

**Fig. 4:**
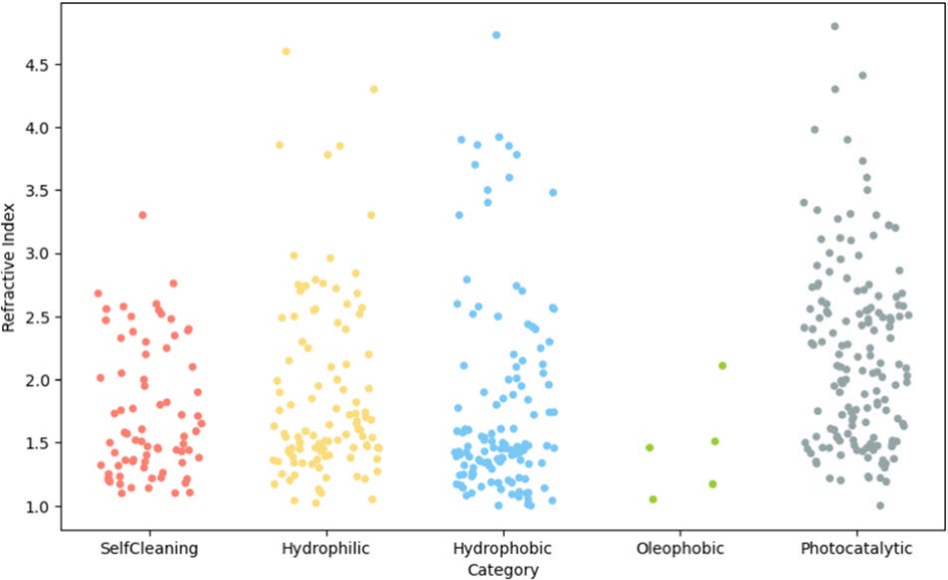
Refractive index distributions of SelfCleaning, Hydrophilic, Hydrophobic, Oleophobic and Photocatalytic datasets.

Figure 4 presents the refractive index distributions for five different datasets. The refractive indices predominantly span the range of 1 to 4, which aligns well with both experimental observations and theoretical predictions for commonplace materials considering their optical bandgaps^32^.

**Fig. 5:**
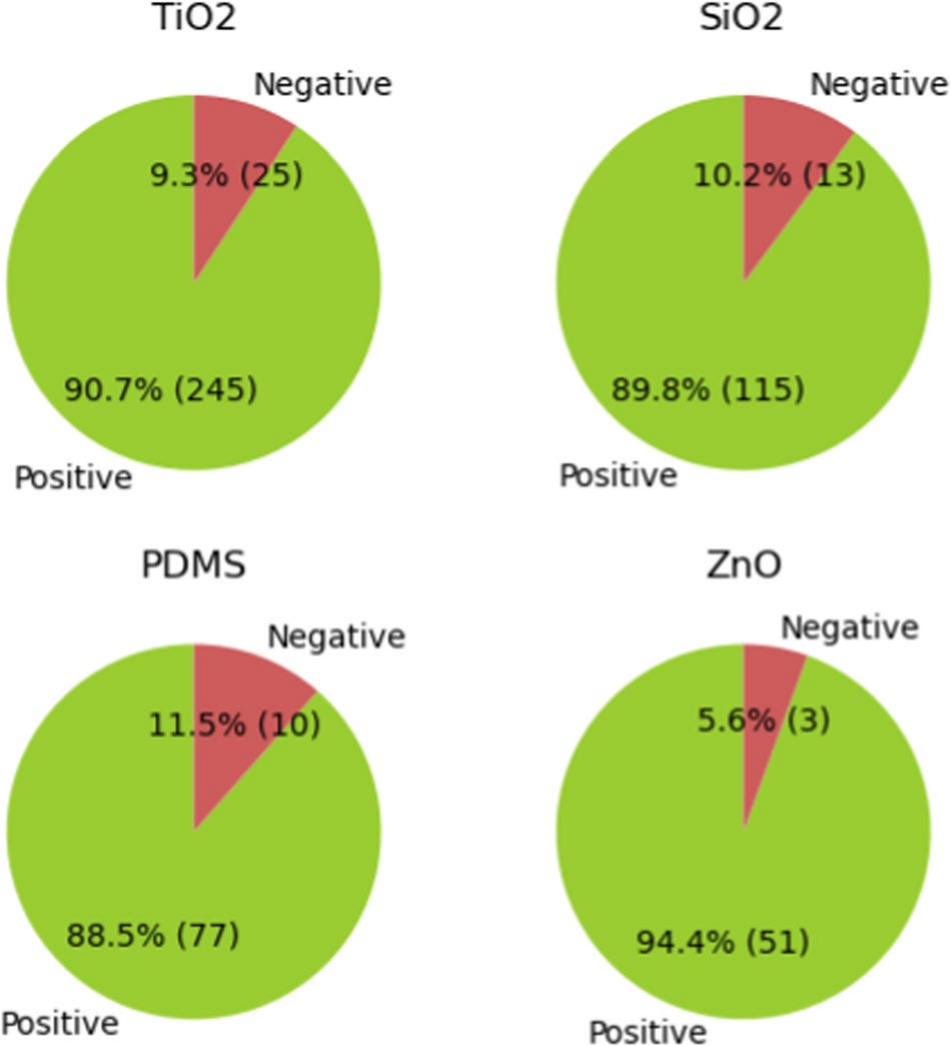
Sentiment results of materials with more than 50 subjective comments in SelfCleaning dataset.

Figure 5 illustrates the materials that have garnered over 50 subjective comments within SelfCleaning sentiment dataset for durability and stability. The top four materials displayed in this figure corroborate with the frequency rank in material name datasets, as materials with a broader research focus tend to accumulate more subjective remarks in the literature. TiO_2_, SiO_2_, and PDMS exhibit similar sentiment results in durability and stability, each with roughly 90% of positive comments. ZnO, however, demonstrates an even higher positive sentiment rate at 94.4%. This can be attributed to the fact that, although ZnO has a relatively short history as a self-cleaning material, its superior performance has garnered considerable attention within a few years. The prominence of ZnO within the self-cleaning literature can be traced back to 2017^33^. Factors such as ZnO’s ease of modification^33^ may explain its elevated positive sentiment rate in durability and stability, as a relatively new promising material.

## Data Availability

The source code used to generate the datasets is available at https://github.com/MasterAI-EAM/self-cleaning. Other codes used are available at https://github.com/MasterAI-EAM/SciCrawler, https://github.com/MasterAI-EAM/TextMaster and https://github.com/CambridgeMolecularEngineering/chemdataextractor2.
